# Are There Any Ethnic Differences in the Response to Baricitinib for the Treatment of Rheumatoid Arthritis?

**DOI:** 10.7759/cureus.20024

**Published:** 2021-11-29

**Authors:** Wajith Hussain Zahir Hussain, Ameen Jubber, Arumugam Moorthy

**Affiliations:** 1 Department of Rheumatology, University Hospitals of Leicester NHS Trust, Leicester, GBR; 2 College of Life Sciences, University of Leicester, Leicester, GBR

**Keywords:** drug response, jak inhibitors, baricitinib, ethnicity, rheumatoid arthritis

## Abstract

Introduction

Baricitinib is an oral synthetic Janus Kinase inhibitor that inhibits JAK1 and JAK2, and the new kid on the block in the treatment of rheumatoid arthritis (RA). To date, there are no studies comparing the clinical benefit of baricitinib in RA between different ethnicities. Ethnicity plays a role in the effectiveness of therapeutic agents. Given the large multi-ethnic population of Leicestershire in the United Kingdom and the range of new therapeutics in RA, we reviewed our cohort of patients with RA to see whether there is any difference in baricitinib Disease Activity Score 28 (DAS28) response between the Asian and White cohorts.

Methods

This was a retrospective study. The patients included were those under the care of rheumatology at University Hospitals of Leicester (UHL) with a diagnosis of RA and either receiving baricitinib or had received it in the past. Data was collected using the UHL information technology systems, clinic letters and pharmacy records. In addition to ethnicity, we reviewed patient age, gender, concurrent disease-modifying anti-rheumatic drugs (DMARDs) used, previous biologics used, baseline and post-treatment DAS28, dropout from therapy, baseline biochemical assays (anti-cyclic citrullinated peptide (anti-CCP) and rheumatoid factor (RF) status) and radiographic findings. An independent t-test was used to compare continuous data, and Pearson’s chi-squared test was used to compare categorical data.

Results

A total of 120 patients were included in the analysis, and data were analysed with Portable Format for Analytics (PFA). There was no statistically significant difference in the mean DAS28 at baseline (Asian: 5.17 versus White: 4.65; p-value = 0.107) and post-treatment (Asian: 2.8 versus White: 3.3; p-value = 0.404). Comparing both ethnicities, there was no statistically significant difference in previous biologics used, anti-CCP and RF titres, and radiographic findings of erosions.

Conclusion

This is the first study of its kind, and it found no significant difference in baricitinib response between the Asian and White cohorts. Our study had certain limitations, and future studies will be needed to evaluate this subject further. Such data is important as it can contribute to a body of evidence that may in the future help inform clinical decision-making.

## Introduction

Baricitinib is an oral synthetic inhibitor of the Janus-associated tyrosine kinases JAK1 and JAK2 used in the management of rheumatoid arthritis (RA). Over 100,000 patients with RA worldwide have been treated with baricitinib to date, and it is approved in over 70 countries for the treatment of RA in adults with moderate to severe disease [1]. It was shown in the RA-BEGIN study to have superior efficacy (both as monotherapy and combination therapy with methotrexate (MTX)) to methotrexate monotherapy [2]. The RA-BEAM study demonstrated that in patients with RA with an inadequate response to methotrexate (MTX), baricitinib led to a significant clinical improvement compared to placebo and adalimumab [3].

Despite ample evidence supporting the benefit of baricitinib in RA, there is a lack of data comparing drug response in different ethnicities. The large multi-ethnic population of Leicester in the United Kingdom presents a unique opportunity in this regard. With a city population of 329,839 and a county population of 650,489 (according to the 2011 census), the two ethnicities that make up the largest share are White (50.52%) and Asian (37%), and this population is served by a large tertiary centre hospital. Of the White population, 95% were White British, and of the Asian population, 85% reported being of Indian heritage. However, we note that these demographic proportions and the total populations may have altered significantly in the last 10 years.

Since baricitinib was approved by the National Institute of Health and Care Excellence (NICE) in 2017, we have noted a surge in the use of this drug in our rheumatology department; around 120 patients to date have received baricitinib for RA. We anticipate that this will continue to rise owing to its acceptable safety profile [4] and its oral formulation that makes it an attractive option for many patients averse to injection therapy.

It is becoming increasingly important for disease-modifying therapy to be tailored to individual patients. The factors that must be taken into account include the patient’s lifestyle, comorbidities, preferred route of administration, whether the drug can be given as monotherapy, infection risk factors (e.g., tuberculosis reactivation), patient fertility and plans for conception and pregnancy [5]. From this perspective, data on drug response between different racial and ethnic groups can contribute to a body of evidence that informs clinical decision-making - something especially pertinent given the growing myriad of treatment options available for patients with RA.

## Materials and methods

Study design and patients

This was a retrospective study. The patients included were those under the care of rheumatology at UHL with a diagnosis of RA and either receiving baricitinib or had received it in the past. This included patients on baricitinib monotherapy and those on baricitinib combination therapy with another disease-modifying antirheumatic drug (DMARD). All patients had an established diagnosis of RA made by a rheumatologist and fulfilled the NICE criteria for commencing baricitinib. Patients who were prescribed baricitinib but never took the drug due to choice were excluded from the study.

Data collection and outcome measures

Data were collected using the UHL electronic medical record system, clinic letters and pharmacy records. In addition to ethnicity, we reviewed patient age, gender, concurrent DMARDs used, previous biologics used, baseline and post-treatment Disease Activity Score 28 (DAS28), dropout from therapy, baseline biochemical assays (anti-cyclic citrullinated peptide (anti-CCP) and rheumatoid factor (RF) status) and the presence of baseline radiographic erosions.

Each patient’s ethnicity was recorded on the electronic medical record system. These ethnic categories were the same as those used in the United Kingdom census, and each patient self-reported their ethnic group. In this study, the term Asian refers only to those of South Asian heritage, originating from the Indian subcontinent. There were no East Asians amongst the patients with RA on baricitinib, and no ethnic groups were excluded from the study.

Statistical analysis

**Figure 1:**
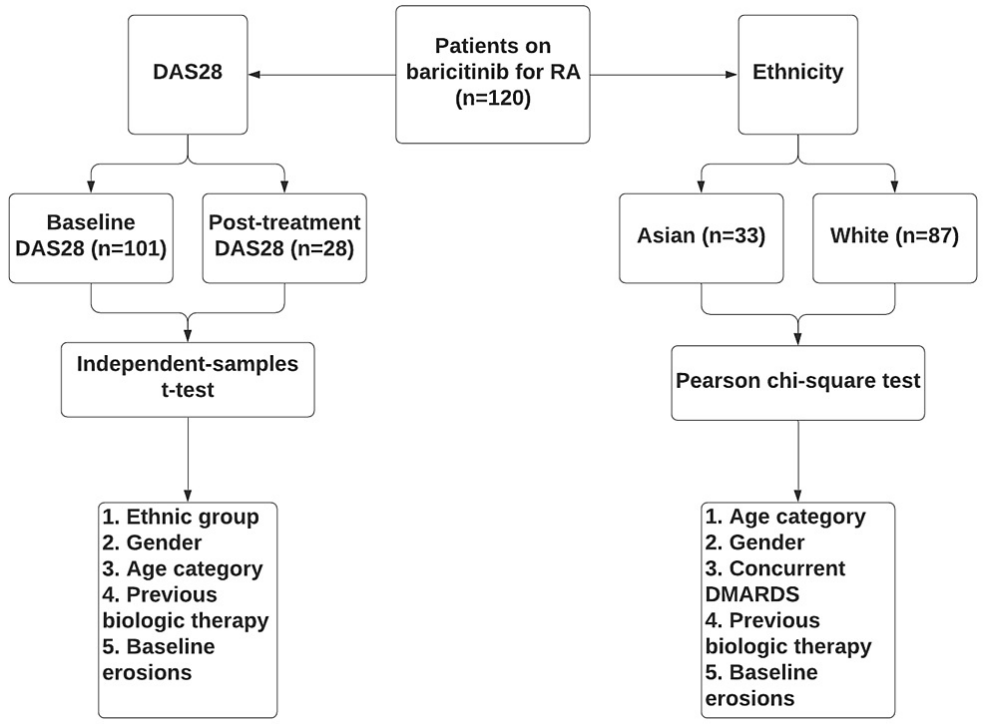
Method of statistical comparison RA = rheumatoid arthritis n = number DAS28 = Disease Activity Score 28 DMARDs = disease-modifying anti-rheumatic drugs

Data were analysed with Portable Format for Analytics (PFA). The independent samples t-test was used to compare continuous data, and Pearson’s chi-squared test was used to compare categorical data (Figure 1).

## Results

In total, 120 patients were included in the analysis (n = 120). White patients made up 72.5% of the patient cohort, with Asian patients making up the remaining 27.5%. These values did not match the demographic data on the 2011 census (White: 50.52%; Asian: 37%).

As expected in most autoimmune diseases, female patients were the majority in both ethnic cohorts. However, the female-to-male ratio in the Asian group was significantly greater (9.99:1) than that in the White group (3.59:1). The average age was higher in the Asian group (mean age: 61.3 years) than in the White group (mean age: 55.15 years).

**Figure 2:**
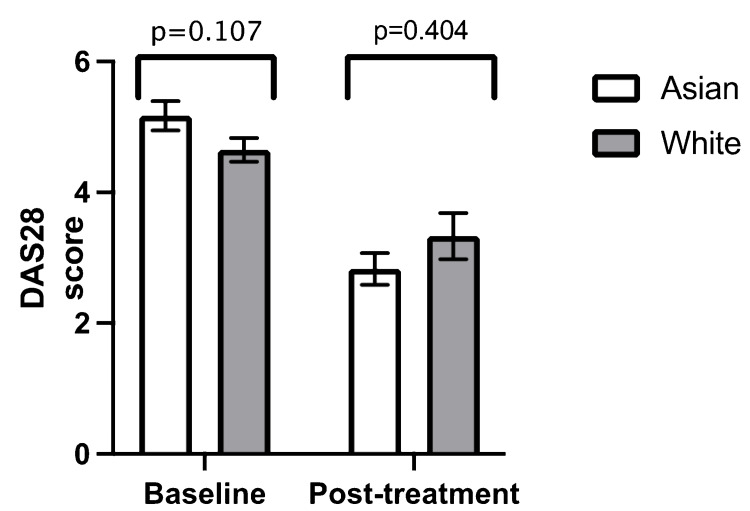
Comparison between mean baseline DAS28 and mean post-treatment DAS28 in ethnicity Error bars = standard error of the mean DAS28 = Disease Activity Score 28

DAS28 at baseline was higher in the Asian patients by 0.52 points, suggesting more aggressive baseline disease, but the p-value was 0.107. Although the mean decline in DAS28 with baricitinib was greater in the Asian group (2.59) compared with the White group (1.25), this was not statistically significant (p-value = 0.07). There was no statistically significant difference in the mean DAS28 at baseline (Asian: 5.17 versus White: 4.65; p-value = 0.107) and post-treatment (Asian: 2.8 versus White: 3.3; p-value = 0.404) (Figure 2) (Table 1).

**Table 1:** Independent samples t-test for ethnicity n = number DAS28 = Disease Activity Score 28 Anti-CCP = anti-cyclic citrullinated peptide RF = rheumatoid factor SD = standard deviation

	White (n = 87)			Asian (n = 33)			
	Mean value	n	SD	Mean value	n	SD	p-value
Baseline DAS28	4.65	71	1.55	5.17	30	1.21	0.107
Post-treatment DAS28	3.33	19	1.53	2.83	8	0.69	0.404
Duration from baseline to post-treatment DAS28 (months)	7.53	19	6.60	9.25	8	6.92	0.545
Anti-CCP (U/mL)	157.39	69	155.77	194.97	29	147.46	0.271
RF (IU/mL)	124.48	77	193	169.44	32	282.34	0.339

In this analysis, patients were not classified as having seropositive or seronegative disease; rather, anti-CCP and RF titres were recorded. There was no statistically significant difference between the White and Asian cohorts in these biochemical assays.

**Figure 3:**
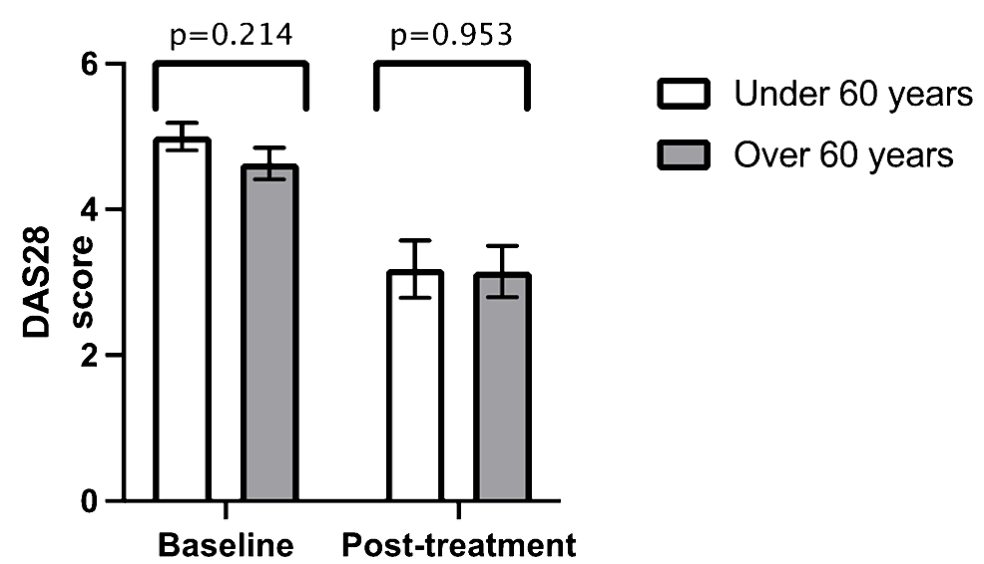
Comparison between mean baseline DAS28 and mean post-treatment DAS28 in age Error bars = standard error of the mean DAS28 = Disease Activity Score 28

**Figure 4:**
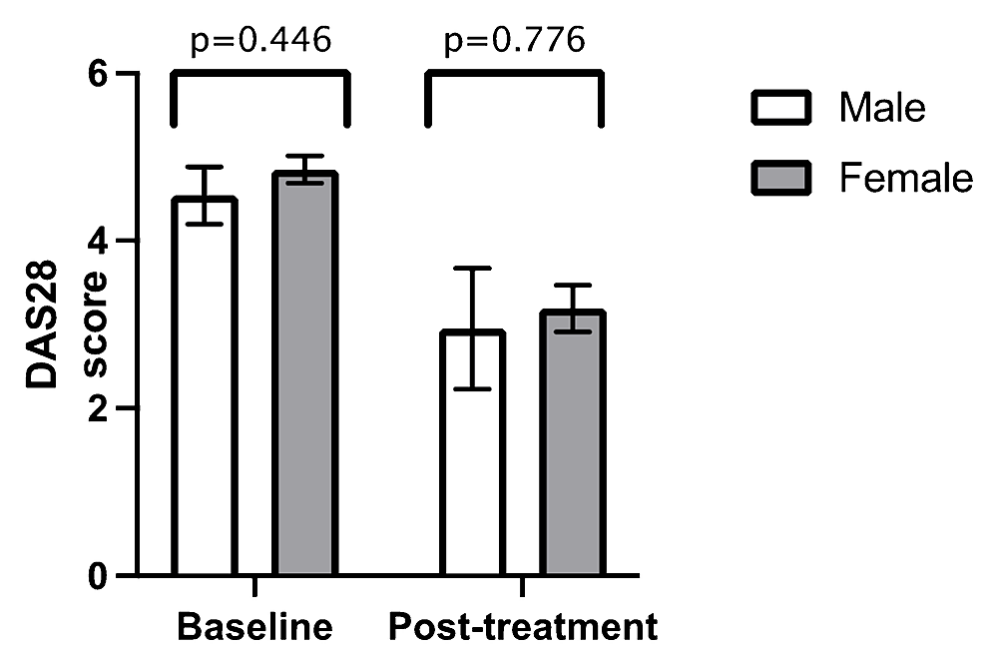
Comparison between mean baseline DAS28 and mean post-treatment DAS28 in gender Error bars = standard error of the mean DAS28 = Disease Activity Score 28

**Figure 5:**
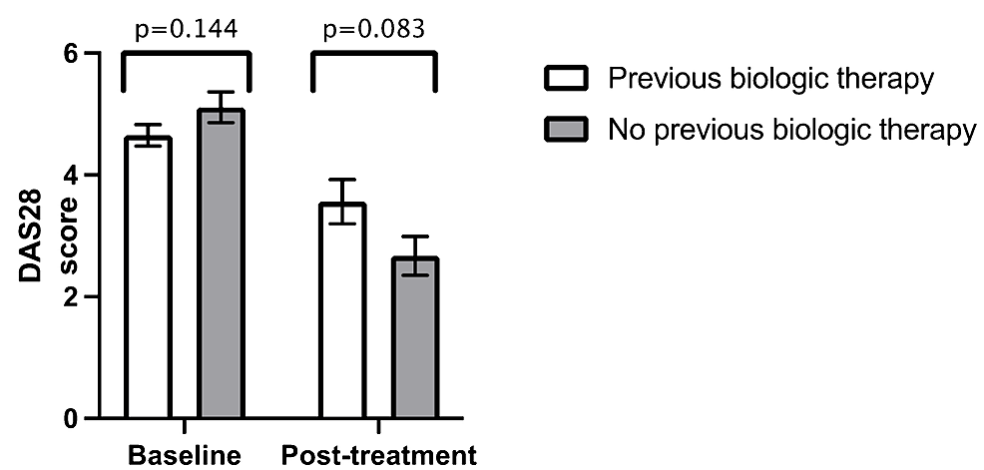
Comparison between mean baseline DAS28 and mean post-treatment DAS28 in previous biologic therapy Error bars = standard error of the mean DAS28 = Disease Activity Score 28

**Figure 6:**
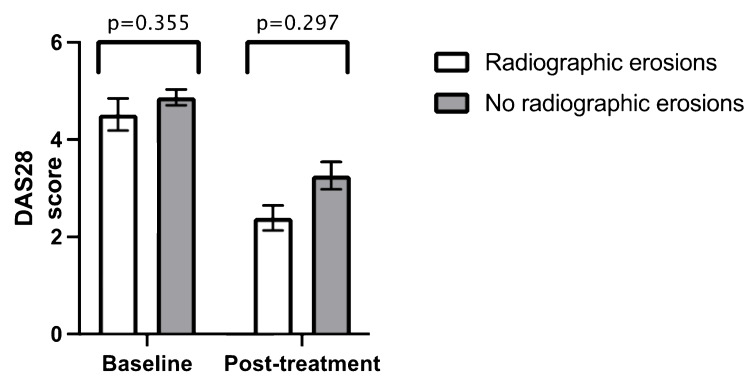
Comparison between mean baseline DAS28 and mean post-treatment DAS28 in radiographic erosions Error bars = standard error of the mean DAS28 = Disease Activity Score 28

Similar to ethnicity, there was no statistically significant difference in mean DAS28 at baseline and post-treatment when comparing age groups (age > 60 years versus age < 60 years) (Figure 3) (Table 2), gender (Figure 4) (Table 3), previous biologic therapy (Figure 5) (Table 4) and the presence of radiographic erosions at baseline (Figure 6) (Table 5).

**Table 2:** Independent samples t-test for age n = number DAS28 = Disease Activity Score 28 SD = standard deviation

	Age < 60 (n = 55)			Age > 60 (n = 65)			
	Mean value	n	SD	Mean value	n	SD	p-value
Baseline DAS28	5.00	46	1.26	4.63	55	1.61	0.214
Post-treatment DAS28	3.18	13	1.41	3.149	14	1.32	0.953
Duration from baseline to post-treatment DAS28 (months)	8.62	13	7.78	7.50	14	5.50	0.669

**Table 3:** Independent samples t-test for gender n = number DAS28 = Disease Activity Score 28 SD = standard deviation

	Male (n = 22)			Female (n = 98)			
	Mean value	n	SD	Mean value	n	SD	p-value
Baseline DAS28	4.54	16	1.37	4.85	85	1.49	0.446
Post-treatment DAS28	2.95	3	1.25	3.19	24	1.37	0.776
Duration from baseline to post-treatment DAS28 (months)	3.00	3	1.00	8.67	24	6.73	0.164

**Table 4:** Independent samples t-test for previous biologic therapy n = number DAS28 = Disease Activity Score 28 SD = standard deviation

	Previous biologics therapy (n = 73)			No previous biologic therapy (n = 47)			
	Mean value	n	SD	Mean value	n	SD	p-value
Baseline DAS28	4.65	68	1.46	5.11	33	1.46	0.144
Post-treatment DAS28	3.56	15	1.41	2.67	12	1.10	0.083
Duration from baseline to post-treatment DAS28 (months)	8.87	15	7.62	7.00	12	5.17	0.475

**Table 5:** Independent samples t-test for radiographic erosions n = number DAS28 = Disease Activity Score 28 SD = standard deviation

	Radiographic erosions (n = 22)			No radiographic erosions (n = 98)			
	Mean value	n	SD	Mean value	n	SD	p-value
Baseline DAS28	4.52	19	1.44	4.87	82	1.48	0.355
Post-treatment DAS28	2.39	3	0.45	3.26	24	1.39	0.297
Duration from baseline to post-treatment DAS28 (months)	6.00	3	5.57	8.29	24	6.77	0.58

Pearson’s chi-squared test was used on the variables (Table 6). Based on the p-values, we can infer that ethnicity and age category (p-value = 0.238), gender (p-value = 0.107), previous biologics used (p-value = 0.385), concurrent DMARDs (p-value = 0.633) and the presence of baseline radiographic erosions (p-value = 0.979) are independent variables.

**Table 6:** Pearson’s chi-squared test DMARDs = disease-modifying anti-rheumatic drugs

Variables tested	Value	Degrees of freedom	Asymptotic significance (two sided)
Ethnicity * age	1.392(b)	1	0.238
Ethnicity * gender	2.597(b)	1	0.107
Ethnicity * previous biologics	0.755(b)	1	0.385
Ethnicity * concurrent DMARDs	0.228(b)	1	0.633
Ethnicity* baseline radiographic erosions	0.001(b)	1	0.979

## Discussion

Previous studies have made important observations in the field of race and ethnicity in rheumatology, documenting the rates of negative beliefs amongst different ethnicities to biologic therapy, and the impact this has on drug adherence [6], as well as rates of drug discontinuation between the different ethnicities [7]. Greater delays in diagnosis have also been noted in patients of South Asian origin [8]. Such data is important for clinicians that aim to practice holistically. However, there is a paucity of data on race and ethnicity in randomised control trials related to RA therapy [9]. The reasons for this will vary by region [10], but one posited in the United States is the lack of sizeable cohorts of non-White ethnicities in trials [11]. This issue became especially pronounced during the COVID-19 pandemic [12].

Any observed difference in drug response between ethnic groups could be due to the differences in the ways those drugs are metabolised (e.g., in the case of methotrexate) [13]; this can be due to genetic or non-genetic reasons [14]. It is also possible that the immune-mediated mechanisms of RA vary between racial groups [15], affecting the impact certain disease-modifying drugs have on disease activity. Indeed, previous studies have observed differences in disease expression between the races in RA [16-20], although separating genetic factors from confounding lifestyle factors, diet, vitamin D levels [21], education and socio-economic factors has proven challenging.

Baricitinib was selected for this study because of the significant rise we have observed in clinicians and patients opting for this therapy since 2017. This may be due to many patients’ preference for tablets rather than injection therapy and less fear about side effects. However, it is not known whether this has any impact on drug adherence.

We compared the baseline and post-treatment DAS28 scores for the White and Asian cohorts receiving baricitinib for RA cohorts in Leicestershire, a multi-ethnic region in the United Kingdom. Further data were also collected to provide more clinical and demographic details on these cohorts. Our results showed no statistically significant difference in the mean baseline and post-baricitinib DAS28 scores between these two groups.

In addition to ethnicity, we also compared different age groups, males and females, whether the patients had received previous biologic therapy and whether there were radiographic erosions at baseline. We found no statistically significant difference in mean baseline and post-baricitinib DAS28 scores between these two different groups. Interestingly, there was only one case of baricitinib dropout after one year of therapy in a patient from the White group.

This study had certain limitations. Firstly, post-treatment DAS28 availability was limited in both ethnic cohorts; this reduced the study sample size. Our data also did not include comorbidity, smoking status, time from diagnosis of RA, patient weight, renal function and whether previous biologic cessation was due to intolerance, or primary or secondary failure. It is also important to note that racial and ethnic classifications are often arbitrary, not clear-cut and based on self-reporting by patients. Other factors also frequently confound the interpretation of findings related to race and ethnicity, such as socio-economic status, access to healthcare within a certain region and lifestyle factors. Furthermore, DAS28 may be limited in its ability to completely capture RA disease activity; patients with chronic pain and RA-related joint damage may have significantly higher scores due to higher tender joint counts and visual analogue scores.

## Conclusions

Baricitinib has become a popular treatment for RA in recent years. Its oral formulation and proven efficacy have made it an attractive option for patients and clinicians alike. As its use continues to rise across the world, it is important to ask whether different racial and ethnic groups can expect the same clinical response. We noted that such data is scarce in the literature. This study had certain limitations, and future larger group matched prospective studies will be needed to evaluate this important subject further.
